# Cognitive Status & Dementia Severity in CVLT-II-SF Forced Choice Recognition: Implications for Effort Assessment

**DOI:** 10.1093/geroni/igab046.2473

**Published:** 2021-12-17

**Authors:** Karl Grewal, Michaella Trites, Megan O'Connell, Andrew Kirk, Stuart MacDonald, Debra Morgan

**Affiliations:** 1 University of Saskatchewan, Saskatoon, Saskatchewan, Canada; 2 University of Victoria, Victoria, British Columbia, Canada

## Abstract

Effort testing is critical to neuropsychological practice, including dementia assessment. Questions exist around whether cognitive status or impairment severity impacts effort test performance in this population. Presently, we examined whether scores on an embedded effort test - the California Verbal Learning Test II Short Form (CVLT-II-SF) Forced Choice Recognition (FCR) - differed across diagnostic cognitive status groups and how severity of impairment modulated test performance. In a sample of memory clinic patients, three cognitive status groups were identified: subjective cognitive impairment (SCI; n = 92), amnestic mild cognitive impairment (a-MCI; n = 18), and dementia due to Alzheimer’s Disease (AD; n = 70). Significant group differences in FCR performance were observed using one-way ANOVA (p < .001), with post-hoc analysis indicating the AD group performed significantly worse scores than the other groups. Using multiple regression, FCR performance was modelled as a function of cognitive status, impairment severity indexed MMSE, and their interaction, with a parallel analysis for the Clinical Dementia Rating Sum of Boxes (CDR-SOB) scores as an alternate severity measure. Results yielded significant main effects for MMSE (p = 0.019) and cognitive status (p = 0.026), as well as a significant interaction (p = 0.021). Thus, increases in impairment severity disproportionately impaired FCR performance for persons with AD, calling into question research-based cut scores for effort determination in dementia contexts. Corresponding CDR-SOB analyses were non-significant. Future research should examine whether CVLT-II-SF-FCR is an appropriately specific inclusion in a best-practice testing battery for evaluating effort in dementia populations.

